# Author Correction: Respiratory immunization using antibiotic-inactivated *Bordetella pertussis* confers T cell-mediated protection against nasal infection in mice

**DOI:** 10.1038/s41564-025-02243-w

**Published:** 2025-12-16

**Authors:** Seyed Davoud Jazayeri, Lisa Borkner, Caroline E. Sutton, Kingston H. G. Mills

**Affiliations:** Immune Regulation Research Group, School of Biochemistry and Immunology, Trinity Biomedical Sciences Institute, Trinity College Dublin, Dublin, Ireland

**Keywords:** Cell vaccines, Bacterial infection

Correction to: *Nature Microbiology* 10.1038/s41564-025-02166-6, published online 10 November 2025.

In the version of the article initially published, in the first paragraph of the Results, chloramphenicol was described as a “beta-lactam antibiotic” but this should have read “broad-spectrum antibiotic”. This error has been corrected in the HTML and PDF versions of the article.

