# Supplementary material for: Respiratory immunization using antibiotic-inactivated Bordetella pertussis confers T cell-mediated protection against nasal infection in mice
Source: Nat Microbiol. 2025 Nov 10;10(12):3094–106. doi: 10.1038/s41564-025-02166-6 (PMC12669047; doi:10.1038/s41564-025-02166-6)
Supplement: Supplementary file 1 — Supplementary Figs. 1–3 and Supplementary Table 1. [file 41564_2025_2166_MOESM1_ESM.pdf]

nature microbiology

Supplementary information

https://doi.org/10.1038/s41564-025-02166-6

Respiratory immunization using antibiotic-inactivated *Bordetella pertussis* confers T cell-mediated protection against nasal infection in mice

In the format provided by the authors and unedited

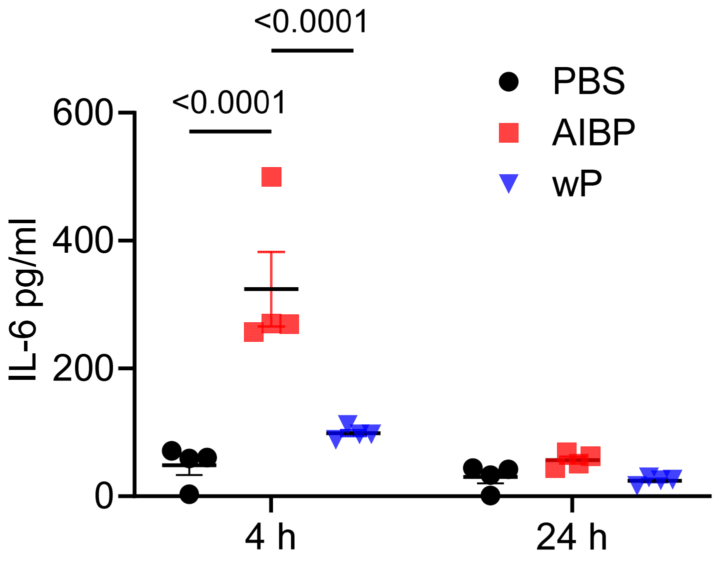

Supplementary Fig. 1. Aerosol administered AIBP vaccine promotes transient induction of IL-6 in the nasal tissue. Mice were aerosol immunized with the AIBP vaccine or were immunized i.m. with a wP vaccine or PBS. After 4 and 24 h, the concentration of IL-6 was quantified in homogenized lung tissue by ELISA. Data are presented as mean ± SEM for biological replicates shown as individual symbols (n = 4). Data were analyzed by two-way ANOVAs followed by Tukey's test for multiple comparisons. P values are shown above relevant data sets.

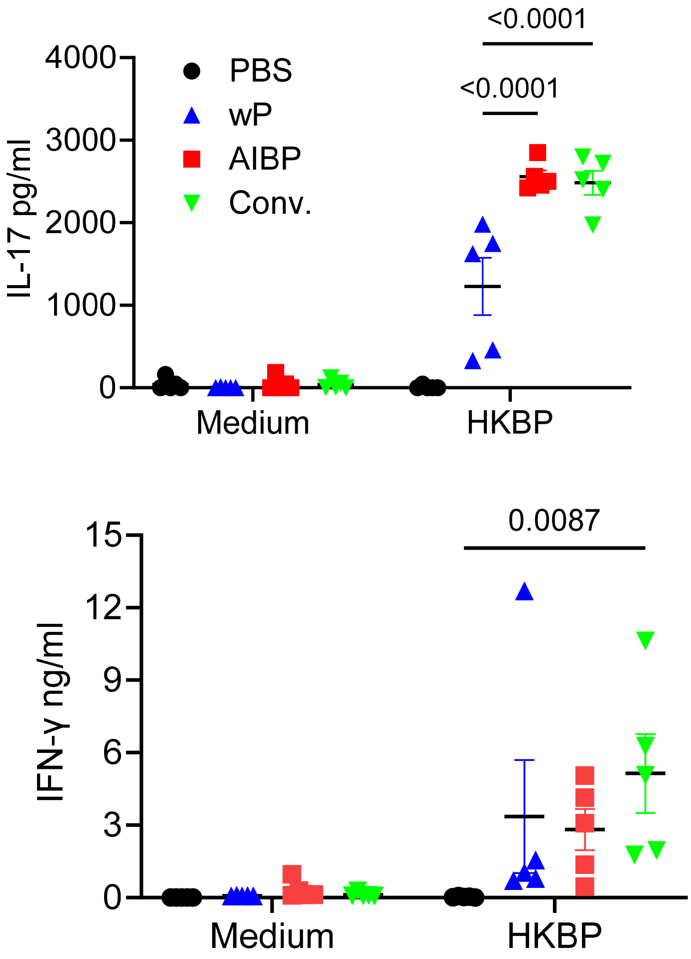

Supplementary Fig. 2. The AIBP vaccine is more effective than a parenterally-delivered wP vaccine at inducing IL-17-secreting T cells in LN and spleen. Mice were immunized by aerosol administration of AIBP vaccine or i.m. administration of a wP vaccine (1/50 of the human dose) or PBS at 0 and 4 weeks or were infected with virulent *B. pertussis* at and allowed to clear the infection. At week 6 single-cell suspensions from cervical and inguinal LN and spleen cells at a ratio of 9:1 (2×10^6/ml) were stimulated with HKBP, or medium only. After 3 days of culture the concentrations of IL-17 and IFN-γ in supernatants were quantified by ELISA. Data are presented as mean ± SEM for biological replicates shown as individual symbols (n = 5). Data were analyzed by one-way ANOVA followed by Tukey's test for multiple comparisons. P values are shown above relevant data sets. .

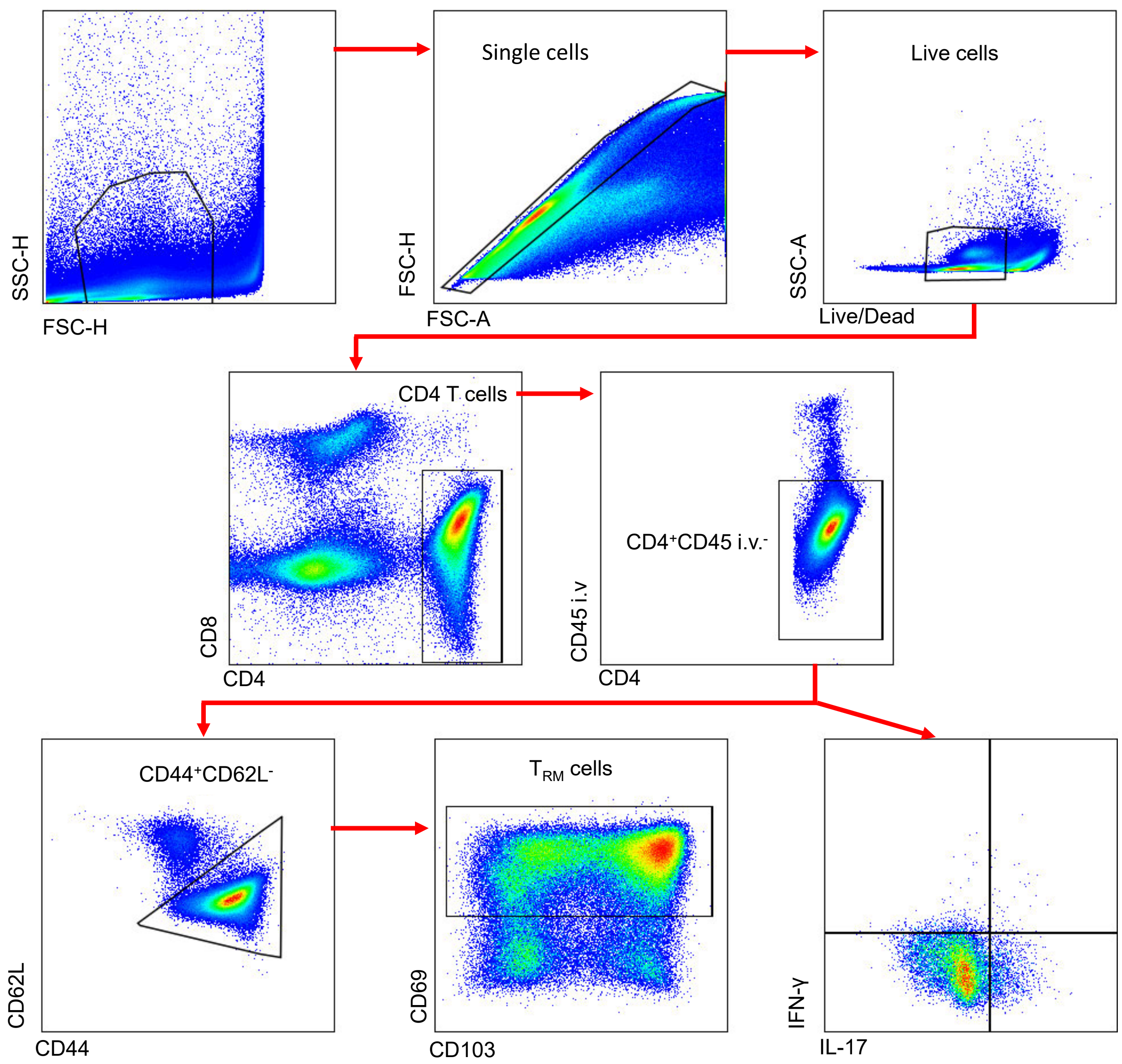

Supplementary Fig. 3. Flow cytometry gating strategy for T cells in respiratory tissues. Gating strategy for identification of CD4 T_{RM} cells ($CD45iv^{-}CD4^{+}CD44^{+}CD62L^{-}$ $CD69^{+}CD103^{+/-}$cells) and, after in vitro re-stimulation with antigen, IL-17A- or IFN-γ-secreting CD4 T_{RM} cells in lung or nasal tissues.

Supplementary Table1. Details of antibodies used in the study

Antibodies (dilution)	Supplier name	Catalog number	Clone name
**Anti-mouse CD69-BB700 (1:100)**	BD Biosciences	566500	H1.2F3
**Anti-mouse CD45R/B220- BV711 (1:200)**	BioLegend	103255	RA3-6B2
**Anti-mouse CD8-APC-eFluor (1:200)**	BD Biosciences	47-0081-82	53-6.7
**Anti-mouse CD3- PE/Cyanine7(1:200)**	BioLegend	100320	145-2C11
**Anti-mouse CD4-PE/Cyanine5 (1:200)**	BD Biosciences	15-0041-82	GK1.5
**Anti-mouse CD44-BV605 (1:600)**	BioLegend	103047	IM7
**Anti-mouse CD62L-PE-CF594 (1:600)**	BD Biosciences	562404	MEL-14
**Anti-mouse CD103-BV786 (1:200)**	BD Biosciences	564322	M290
**Anti-mouse Ly6G- BV421(1:200)**	BioLegend	127628	1A8
**Anti-mouse CD11b- BV711(1:200)**	BioLegend	101242	M1/70
**Anti-mouse IFN-γ-APC (1:200)**	eBiosciences	17-7311-81	XMG1.2
**Anti-mouse IL17-A-FITC (1:200)**	BioLegend	506908	TC11-18H10
**Anti-mouse IL5-BV421 (1:200)**	BioLegend	504311	TRFK5
**Anti-mouse Siglec-F^{+}- eFluor660 (1:200)**	eBiosciences	50-1702-82	1RN44N
**Anti-mouse MHC class II- APC (1:200)**	BioLegend	107614	M5/114.15.2
**Anti-mouse CD80-BV421 (1:200)**	BD Biosciences	562611	16-10A1
**Anti-mouse CD86- PE/Cyanine5 (1:200)**	BioLegend	105016	GL-1
**Anti-mouse CD3-APC (1:80)**	BioLegend	100312	145-2C11
**Anti-mouse CD11b-APC-eFluor780 (1:640)**	eBiosciences	47-0112-82	M1/70
**Anti-mouse CD19- PE/Cyanine5 (1:320)**	BioLegend	115520	6D5
**Anti-mouse CD45-PE (1:640)**	eBiosciences	12-0451-82	30-F11
**Anti-mouse Ly6G-Pacific Blue (1:200)**	BioLegend	127612	1A8
**Anti-mouse Ly6C-FITC (1:800)**	BioLegend	128006	HK1.4
