# Supplementary material for: Respiratory immunization using antibiotic-inactivated Bordetella pertussis confers T cell-mediated protection against nasal infection in mice
Source: Nat Microbiol. 2025 Nov 10;10(12):3094–106. doi: 10.1038/s41564-025-02166-6 (PMC12669047; doi:10.1038/s41564-025-02166-6)
Supplement: Supplementary file 2 — Reporting Summary [file 41564_2025_2166_MOESM2_ESM.pdf]

Corresponding author(s): Kingston Mills, DBPR-NMICROBIOL-25010347

Last updated by author(s): 25 Sept 2025

Reporting Summary

Nature Portfolio wishes to improve the reproducibility of the work that we publish. This form provides structure for consistency and transparency in reporting. For further information on Nature Portfolio policies, see our Editorial Policies and the Editorial Policy Checklist.

Statistics

For all statistical analyses, confirm that the following items are present in the figure legend, table legend, main text, or Methods section.

n/a	Confirmed	
☐	☒	The exact sample size (n) for each experimental group/condition, given as a discrete number and unit of measurement
☐	☒	A statement on whether measurements were taken from distinct samples or whether the same sample was measured repeatedly
☐	☒	The statistical test(s) used AND whether they are one- or two-sided<br>*Only common tests should be described solely by name; describe more complex techniques in the Methods section.*
☐	☒	A description of all covariates tested
☐	☒	A description of any assumptions or corrections, such as tests of normality and adjustment for multiple comparisons
☐	☒	A full description of the statistical parameters including central tendency (e.g. means) or other basic estimates (e.g. regression coefficient) AND variation (e.g. standard deviation) or associated estimates of uncertainty (e.g. confidence intervals)
☐	☒	For null hypothesis testing, the test statistic (e.g. F, t, r) with confidence intervals, effect sizes, degrees of freedom and P value noted<br>*Give P values as exact values whenever suitable.*
☒	☐	For Bayesian analysis, information on the choice of priors and Markov chain Monte Carlo settings
☒	☐	For hierarchical and complex designs, identification of the appropriate level for tests and full reporting of outcomes
☒	☐	Estimates of effect sizes (e.g. Cohen's d, Pearson's r), indicating how they were calculated

Our web collection on statistics for biologists contains articles on many of the points above.

Software and code

Policy information about availability of computer code

Data collection	Not applicable
Data analysis	Statistical analyses were performed using Graph-Pad Prism 9.0 Software.

For manuscripts utilizing custom algorithms or software that are central to the research but not yet described in published literature, software must be made available to editors and reviewers. We strongly encourage code deposition in a community repository (e.g. GitHub). See the Nature Portfolio guidelines for submitting code & software for further information.

Data

Policy information about availability of data

All manuscripts must include a data availability statement. This statement should provide the following information, where applicable:

- Accession codes, unique identifiers, or web links for publicly available datasets
- A description of any restrictions on data availability
- For clinical datasets or third party data, please ensure that the statement adheres to our policy

All data are available on request.

Research involving human participants, their data, or biological material

Policy information about studies with human participants or human data. See also policy information about sex, gender (identity/presentation), and sexual orientation and race, ethnicity and racism.

Reporting on sex and gender	Not applicable
Reporting on race, ethnicity, or other socially relevant groupings	Not applicable
Population characteristics	Not applicable
Recruitment	Not applicable
Ethics oversight	Not applicable

Note that full information on the approval of the study protocol must also be provided in the manuscript.

Field-specific reporting

Please select the one below that is the best fit for your research. If you are not sure, read the appropriate sections before making your selection.

☒ Life sciences ☐ Behavioural & social sciences ☐ Ecological, evolutionary & environmental sciences

For a reference copy of the document with all sections, see nature.com/documents/nr-reporting-summary-flat.pdf

Life sciences study design

All studies must disclose on these points even when the disclosure is negative.

Sample size	Sample sizes were chosen based on findings from similar previous experiments.
Data exclusions	No data were excluded from the analysis.
Replication	Each experiments was carried out 2-3 times with consistent and reproducible results. We have no reason to believe that reproducibility is an issue.
Randomization	C57BL/6 mice (6-8 weeks old) were randomly allocated to experimental groups.
Blinding	Mice were randomly allocated to experimental groups. Experimenters were unaware of the origin of cells and sample during analysis although no formal arrangements were in place.

Reporting for specific materials, systems and methods

We require information from authors about some types of materials, experimental systems and methods used in many studies. Here, indicate whether each material, system or method listed is relevant to your study. If you are not sure if a list item applies to your research, read the appropriate section before selecting a response.

Materials & experimental systems

n/a	Involved in the study	
☐	☒	Antibodies
☒	☐	Eukaryotic cell lines
☒	☐	Palaeontology and archaeology
☐	☒	Animals and other organisms
☒	☐	Clinical data
☒	☐	Dual use research of concern
☒	☐	Plants

Methods

n/a	Involved in the study	
☒	☐	ChIP-seq
☐	☒	Flow cytometry
☒	☐	MRI-based neuroimaging

Antibodies

Antibodies used	The details of antibodies used are provided in the Methods section and in Supplementary Table 1.

Validation	Antibodies were chosen based on manufacturer's website and previous published studies from our lab.

Animals and other research organisms

Policy information about studies involving animals; ARRIVE guidelines recommended for reporting animal research, and Sex and Gender in Research

Laboratory animals	Female mice of the C57BL/6 strain were obtained from Charles River U.K. Mice were 6-8 weeks old at the initiation of experiments and housed in a specific pathogen-free facility in the Comparative Medicine Unit (CMU), Trinity College Dublin.
Wild animals	Not applicable
Reporting on sex	Findings apply to Female mice only.
Field-collected samples	Not applicable
Ethics oversight	All animal experiments were conducted according to the guidelines and under licenses approved by the Health Products Regulatory Authority (HPRA) of Ireland in accordance with prior ethical approval from Trinity College Dublin Animal Research Ethics Committee.

Note that full information on the approval of the study protocol must also be provided in the manuscript.

Plants

Seed stocks	Not applicable
Novel plant genotypes	Not applicable
Authentication	Not applicable

Flow Cytometry

Plots

Confirm that:

☐ The axis labels state the marker and fluorochrome used (e.g. CD4-FITC).

☐ The axis scales are clearly visible. Include numbers along axes only for bottom left plot of group (a 'group' is an analysis of identical markers).

☒ All plots are contour plots with outliers or pseudocolor plots.

☒ A numerical value for number of cells or percentage (with statistics) is provided.

Methodology

Sample preparation	Lungs and nasal tissues were digested and mashed through 70 µm strainer, red blood cells were lysed with ammonium chloride-potassium (ACK) buffer, cells were centrifuged at 1350 rpm, and used for flow cytometric stainig.
Instrument	Flow cytometric analysis was performed on an Aurora.
Software	Data were acquired using SpectroFlo. Data were analysed using FlowJo software (Tree Star).
Cell population abundance	Not applicable
Gating strategy	SSC-H/FSC-H-FSC-H/FSC-A-SSC-S/Live dead-CD8/CD4-CD45iv/CD4+-CD44+/CD62L-CD69+/CD103+-/-IL17-FNg+. Details provided in Supplementary Fig. 3.

☒ Tick this box to confirm that a figure exemplifying the gating strategy is provided in the Supplementary Information.
