# Supplementary figures and images for: Respiratory immunization using antibiotic-inactivated Bordetella pertussis confers T cell-mediated protection against nasal infection in mice
Source: Nat Microbiol. 2025 Nov 10;10(12):3094–106. doi: 10.1038/s41564-025-02166-6 (PMC12669047; doi:10.1038/s41564-025-02166-6)

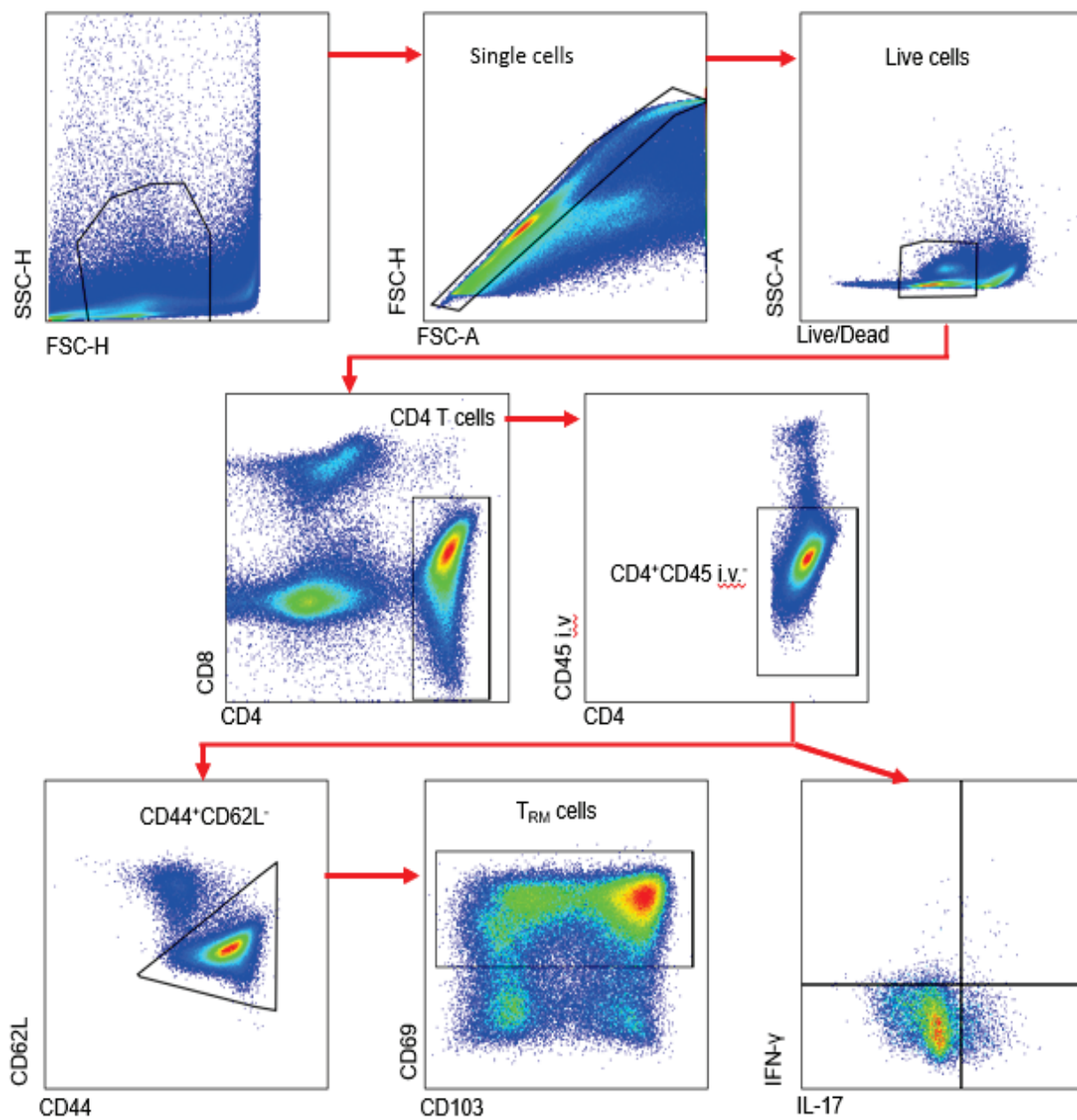

Supplement: Supplementary file 6 — Original flow cytometry plots for Supplementary Fig. 3. [file 41564_2025_2166_MOESM6_ESM.pdf]

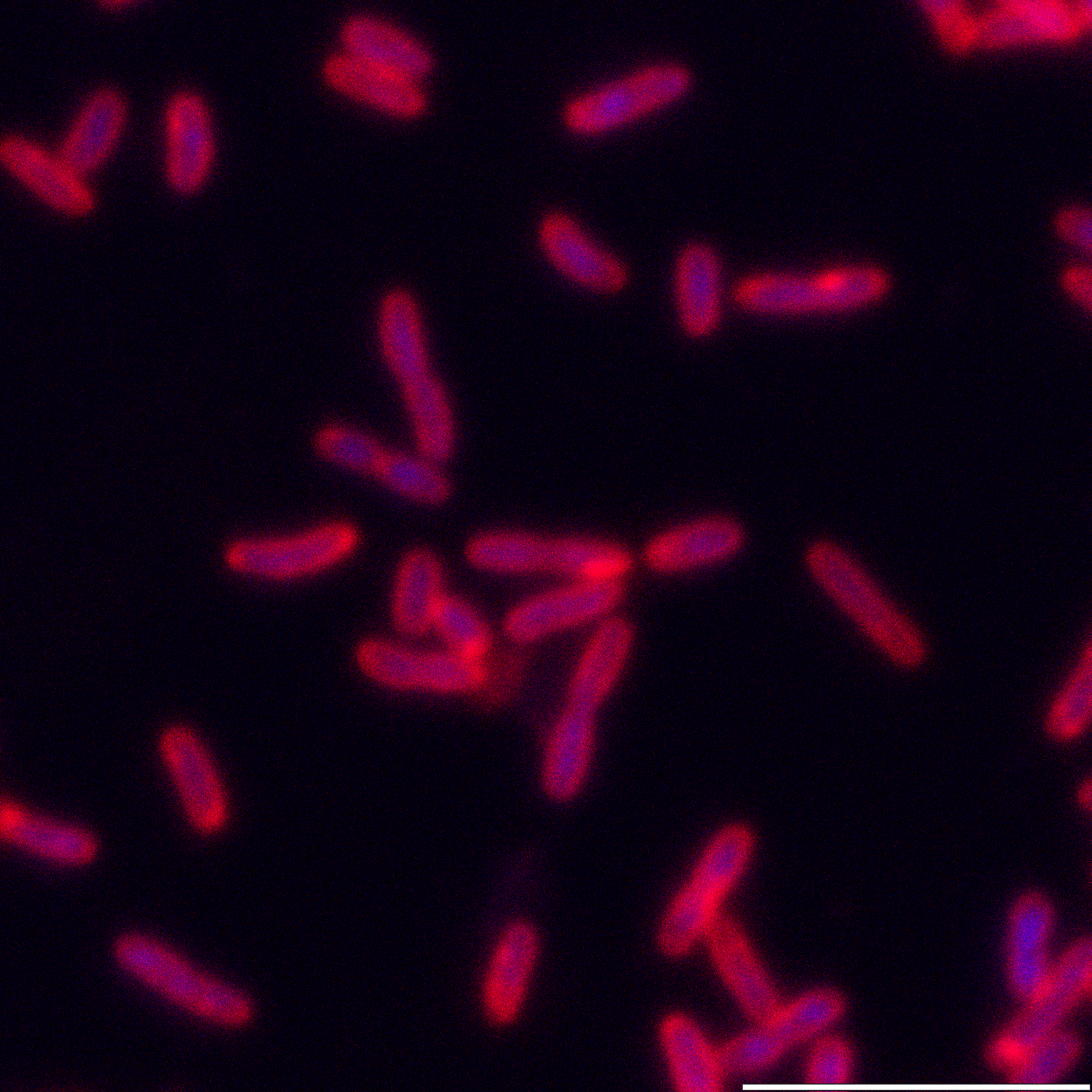

Supplement: Supplementary file 13 — Images of bacteria. [file 41564_2025_2166_MOESM13_ESM.zip › ED Fig.1/DJ_source_data_Extended_data__Figure_1a.tif]

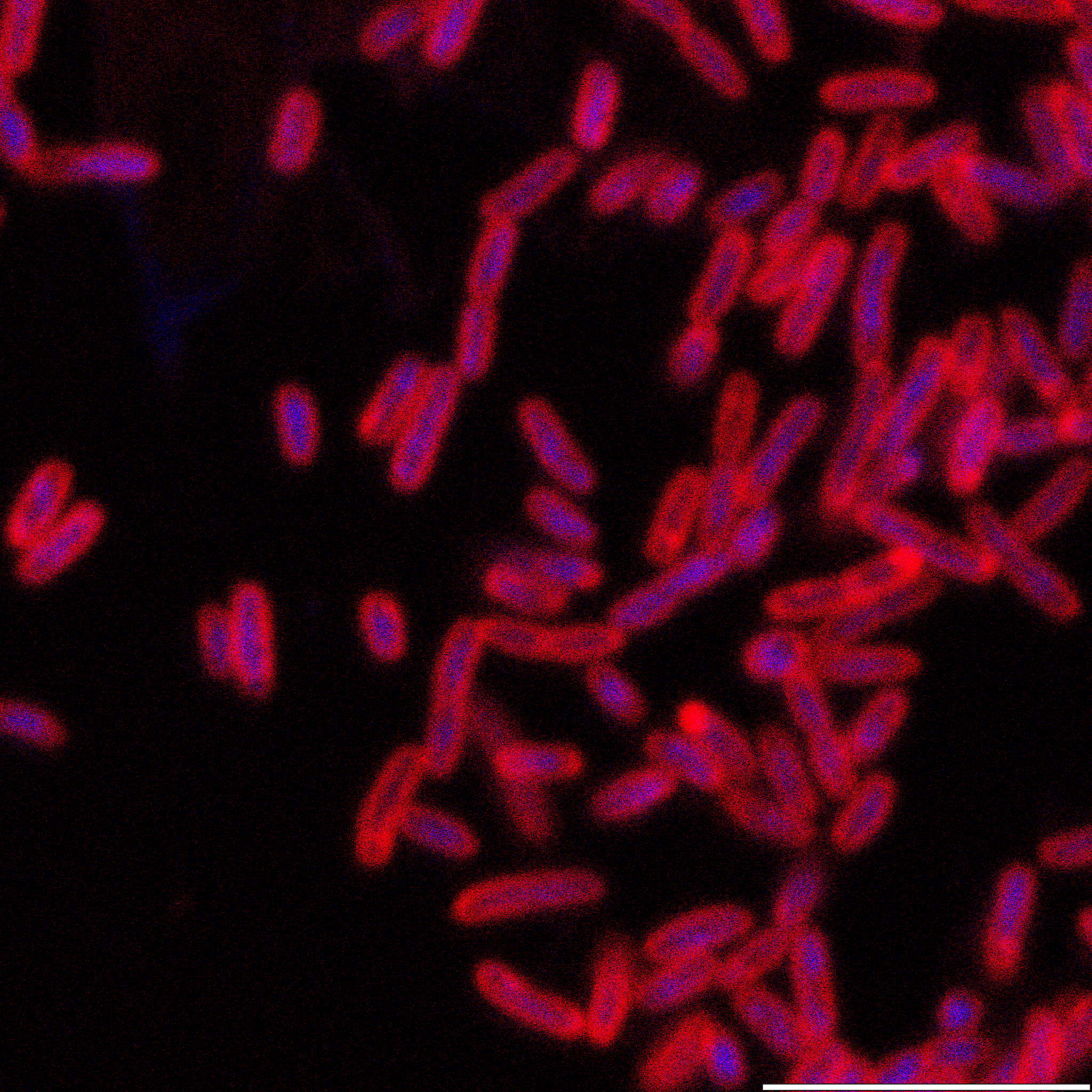

Supplement: Supplementary file 13 — Images of bacteria. [file 41564_2025_2166_MOESM13_ESM.zip › ED Fig.1/DJ_source_data_Extended_data__Figure_1b.tif]

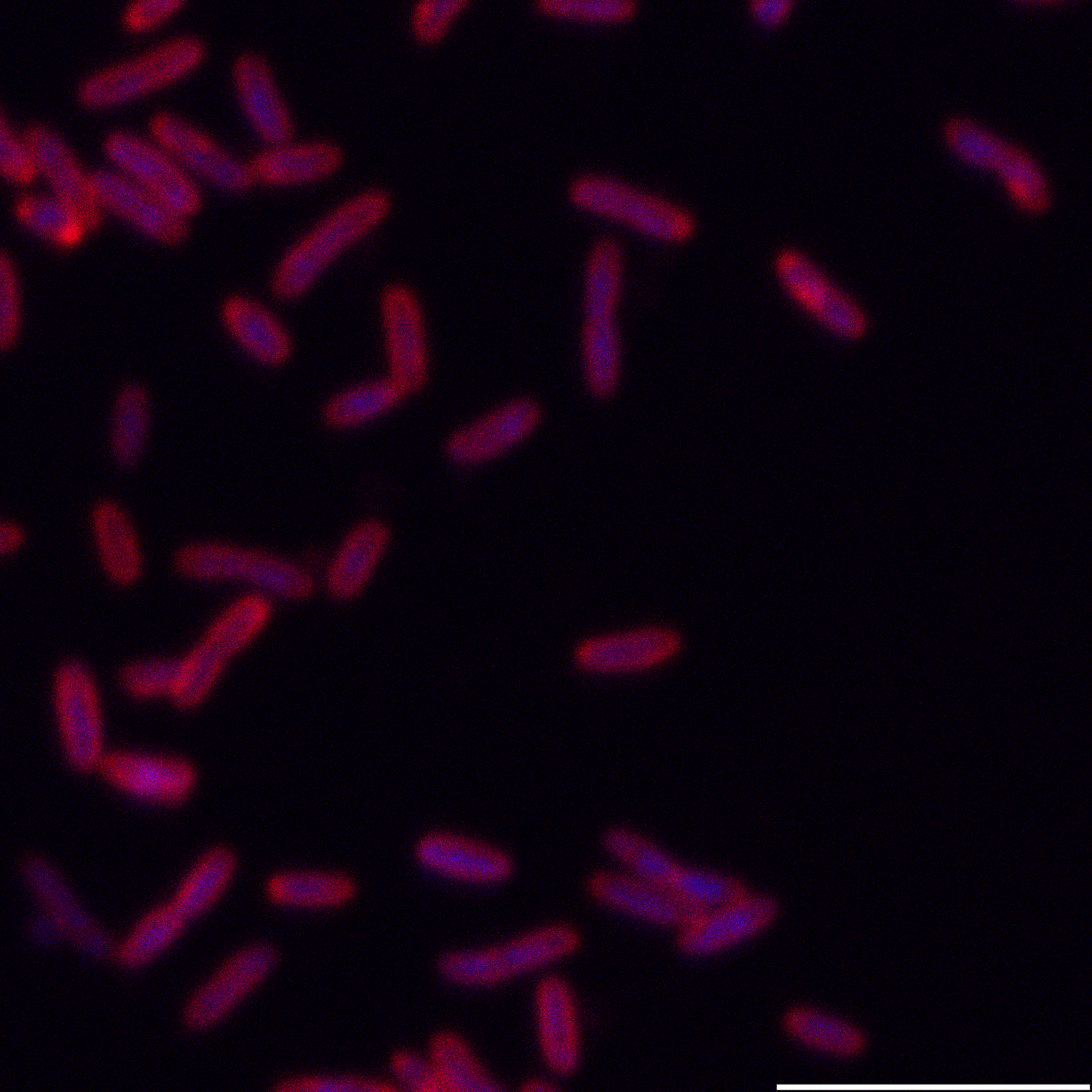

Supplement: Supplementary file 13 — Images of bacteria. [file 41564_2025_2166_MOESM13_ESM.zip › ED Fig.1/DJ_source_data_Extended_data__Figure_1c.tif]

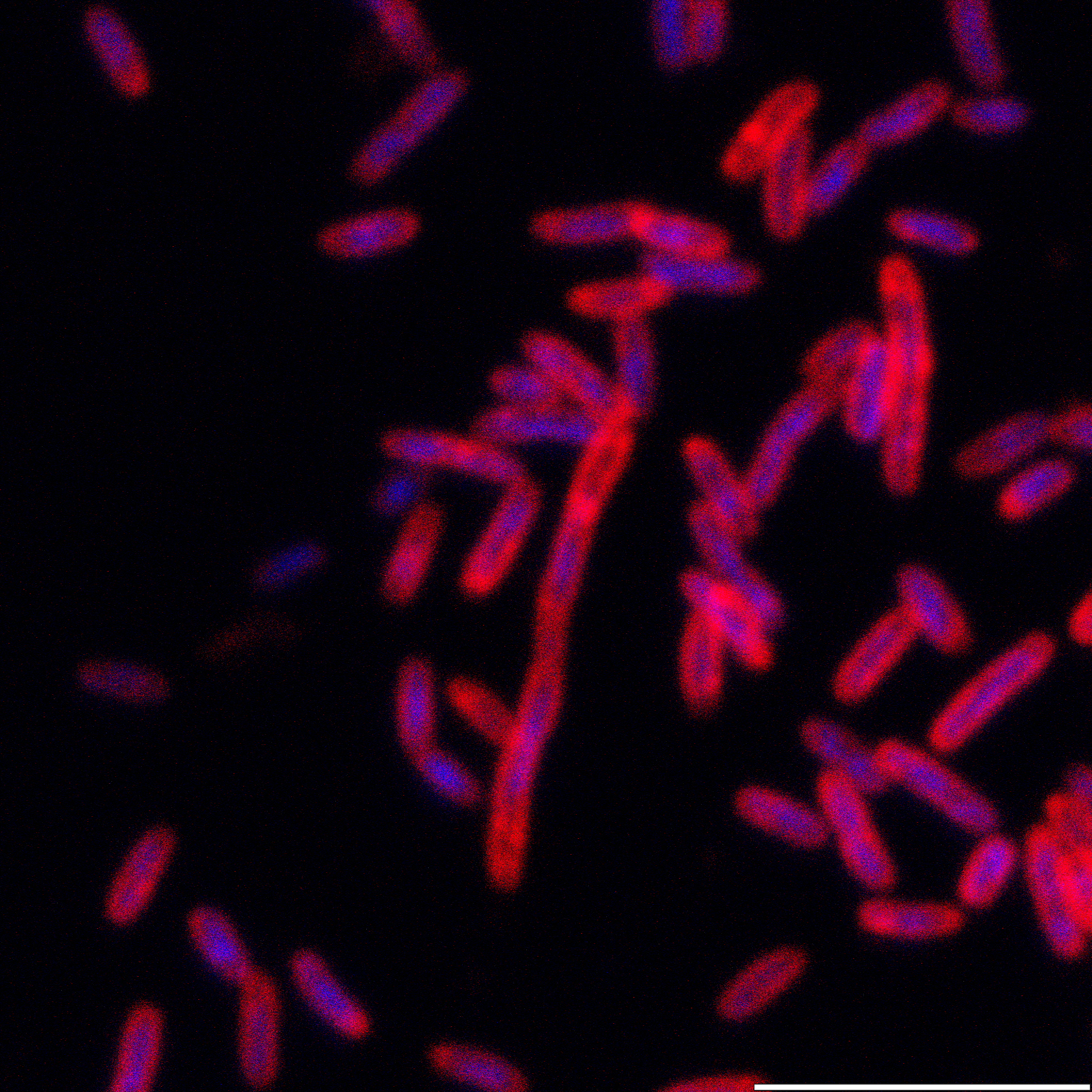

Supplement: Supplementary file 13 — Images of bacteria. [file 41564_2025_2166_MOESM13_ESM.zip › ED Fig.1/DJ_source_data_Extended_data__Figure_1d.tif]

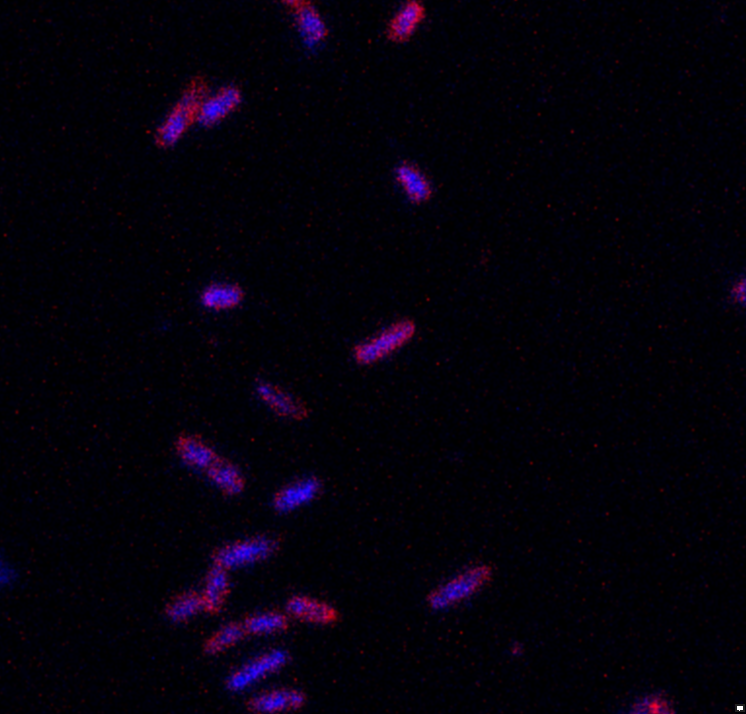

Supplement: Supplementary file 13 — Images of bacteria. [file 41564_2025_2166_MOESM13_ESM.zip › ED Fig.1/DJ_source_data_Extended_data__Figure_1e.tif]

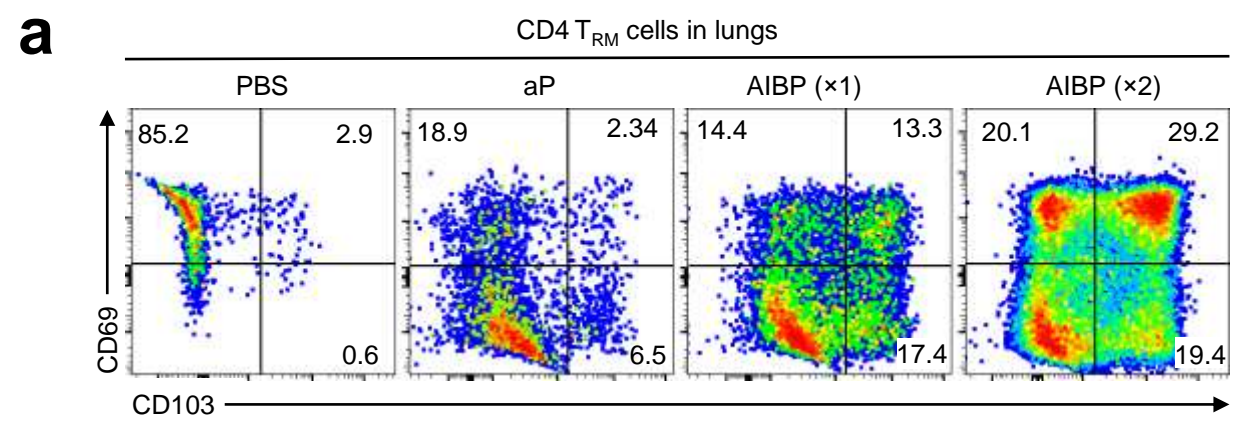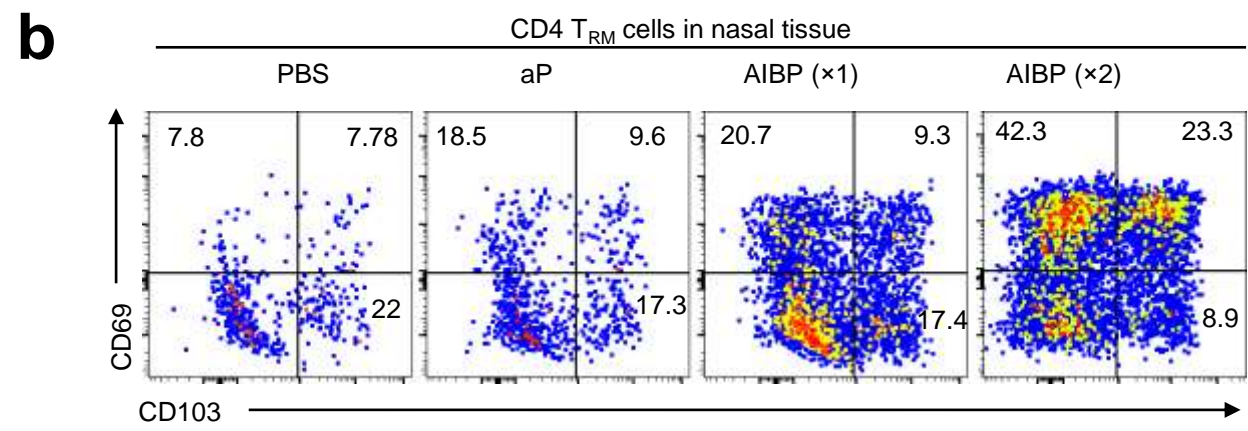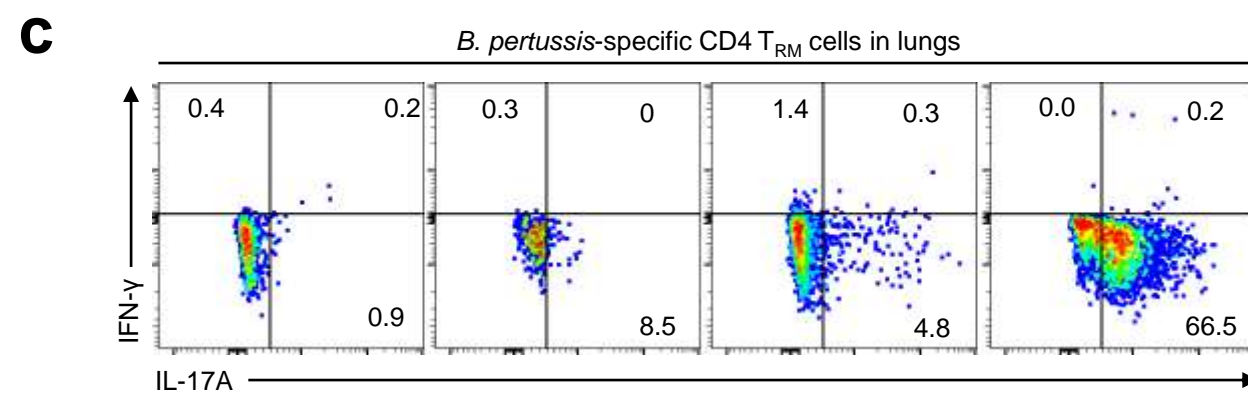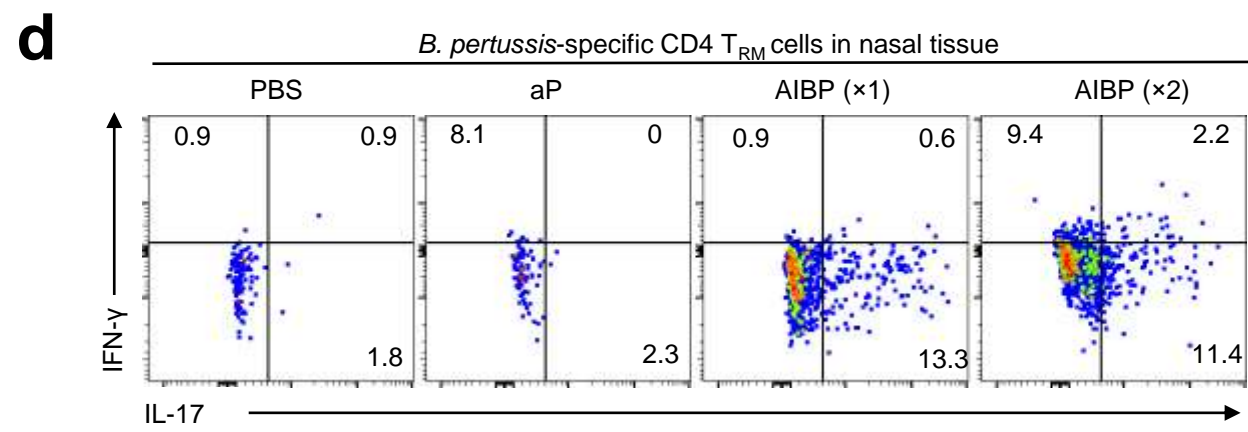

Supplement: Supplementary file 17 — PDF of original flow cytometry plots. [file 41564_2025_2166_MOESM17_ESM.pdf]
